# Serum from calorie-restricted animals delays senescence and extends the lifespan of normal human fibroblasts *in vitro*

**DOI:** 10.18632/aging.100719

**Published:** 2015-01-13

**Authors:** Rafael de Cabo, Lijuan Liu, Ahmed Ali, Nathan Price, Jing Zhang, Mingyi Wang, Edward Lakatta, Pablo M. Irusta

**Affiliations:** ^1^ Experimental Gerontology Section, Translational Gerontology Branch, NIA/NIH, Baltimore, MD 21224, USA; ^2^ Laboratory of Cardiovascular Science and Cardiovascular Function Section, NIA/NIH, Baltimore, MD 21224, USA; ^3^ Department of Human Science, Georgetown University Medical Center, Washington, DC 20057 USA

**Keywords:** human diploid fibroblasts, senescence, caloric restriction, lifespan extension, SIRT1, aging

## Abstract

The cumulative effects of cellular senescence and cell loss over time in various tissues and organs are considered major contributing factors to the ageing process. In various organisms, caloric restriction (CR) slows ageing and increases lifespan, at least in part, by activating nicotinamide adenine dinucleotide (NAD^+^)-dependent protein deacetylases of the sirtuin family. Here, we use an *in vitro* model of CR to study the effects of this dietary regime on replicative senescence, cellular lifespan and modulation of the SIRT1 signaling pathway in normal human diploid fibroblasts. We found that serum from calorie-restricted animals was able to delay senescence and significantly increase replicative lifespan in these cells, when compared to serum from *ad libitum* fed animals. These effects correlated with CR-mediated increases in SIRT1 and decreases in p53 expression levels. In addition, we show that manipulation of SIRT1 levels by either over-expression or siRNA-mediated knockdown resulted in delayed and accelerated cellular senescence, respectively. Our results demonstrate that CR can delay senescence and increase replicative lifespan of normal human diploid fibroblasts *in vitro* and suggest that SIRT1 plays an important role in these processes. (185 words).

## INTRODUCTION

The multifactorial process of ageing is thought to involve the cumulative effects of cell loss in various tissues and organs throughout life due in part to cellular senescence [1–9]. Age-associated cell loss and the consequent generation of hostile microenvironments are believed to render the elderly susceptible to various stresses, e.g. overloading cholesterol and oxidative stress [10–15]. These age-associated metabolic dysfunctions further contribute to fragility of otherwise healthy older cells and organs, exacerbating the ageing process. Normal human diploid somatic cells cultured in the laboratory, recapitulate the senescence process observed in ageing cells by displaying a finite replicative lifespan [3,6,16]. After a limited number of cellular divisions, these cells enter a state of replicative senescence, which is characterized by irreversible growth arrest and secretion of senescence-associated bioactive factors like matrix metalloproteinases (MMPs) [17,18].

In a wide range of organisms ranging from yeast to mammals, calorie restriction (CR) has proven to be a highly reproducible intervention for extending lifespan as well as effectively retarding the onset and reducing the incidence of age-related diseases [19–25]. Several beneficial CR-associated effects are due to decreased levels of oxidative stress and maintained metabolic homeostasis [26–28]. Nicotinamide adenine dinucleo-tide (NAD^+^)-dependent protein deacetylases of the sirtuin family are induced by CR in all experimental systems studied and have been postulated to be at the core of most of these effects [29–32]. In *Saccharomyces cerevisiae*, *Caenorhabditis elegans* and *Drosophila melanogaster* increased Sir2 levels have been directly associated with lifespan extension [33–37] and the mammalian SIRT1 protein is regarded as one of the candidate mediators of the longevity effect of CR in rodents [38–40]. Supporting this notion, it has been shown that over-expression of SIRT1 in transgenic mice results in physiologic responses that resemble CR treatments [41], and pharmacological interventions with molecules that activate SIRT1 (e.g. resveratrol) extend the lifespan of mice fed a high fat diet [42,43].

We have previously described an *in vitro* model of CR using cell cultures grown in medium supplemented with serum from animals on CR diets [44]. Many of the features of CR, including reduced cellular proliferation, enhanced stress responsiveness and changes in gene expression could effectively be reproduced in this system. In particular, CR serum leads to increased SIRT1 protein levels in cultured cells [38]. Thus, several effects of CR appear to be mediated by circulating factors in the sera of the animals subjected to the dietary regimen and can be recapitulated *in vitro*. In this study we investigated the effects of CR on replicative senescence. To this end, we cultured normal human diploid fibroblasts *in vitro* in the presence of serum from rats fed on CR (40%) versus *ad libitum* (AL) diets, and assessed the consequences on replicative capacity, cellular lifespan and modulation of the SIRT1 signaling pathway.

## RESULTS

### CR serum delays the onset of senescent phenotypic changes and extends the replicative lifespan of normal human diploid fibroblasts *in vitro*

**Figure 1:**
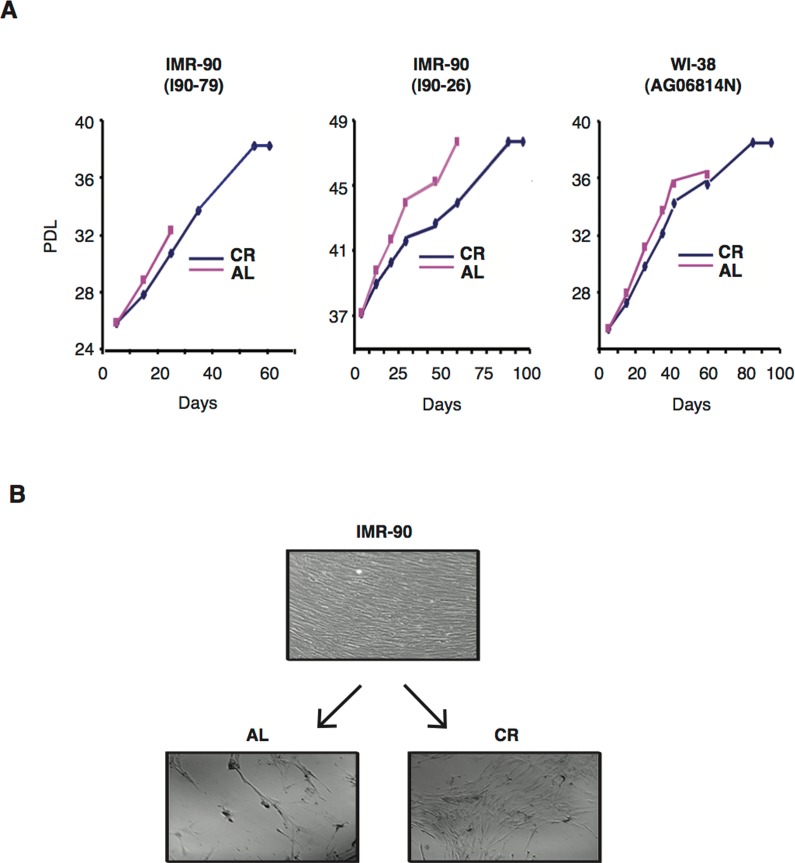
CR serum delays senescence and extends the lifespan of normal human diploid fibroblasts (**A**) Cumulative growth curves of various normal human fibroblast lines cultured in medium containing 10% of either AL (red lines) or CR (blue lines) rat serum. Data obtained with IMR-90 (I90-79) (left panel), IMR-90 (I90-26) (middle panel) and WI-38 (right panel) are shown. PDL, population doublings (**B**) Representative photomicrographs of early passage IMR-90 cells (PDL 25) grown in medium with 10% FBS (upper panel) and after undergoing serial subpassages in the present of AL (left panels) or CR (right panels) rat serums are shown. Original magnification: X100.

Normal human diploid cells grown *in vitro* exhibit a restricted population doubling potential and eventually enter an irreversible growth-arrested state known as replicative senescence, which has been proposed to reflect cellular ageing [45,46]. To determine whether CR can affect senescence entry and lifespan of normal human diploid fibroblasts *in vitro*, three independent normal human fibroblast cell lines were subpassaged in media supplemented with either 10 % CR rat serum or 10% AL rat serum, and analyzed until they reached senescence. Figure 1A shows the growth curves under these conditions for the three cell lines tested, namely IMR-90 (I90-79) (left panel), IMR (I90-26) (middle panel), and WI-38 (AG06814N) (right panel). Exposure of cultured IMR-90 (I90-79) and WI-38 cells to CR serum induced a significant increase in population doubling levels (PDL) and all three cell lines showed a profound delayed in the onset of senescent growth arrest. The average lifespan (cumulative PDL time) of these cells in the presence of CR serum was markedly increased when compared to that of cells grown in medium containing AL serum.

To examine phenotypic changes during serial cultivation, fibroblast morphology was assessed throughout the study. Early passage IMR-90 fibroblasts (PDL 25) grown in 10% FBS displayed a typical spindle-shaped fibroblastic phenotype, grew to confluency, and were contact-inhibited (Fig. 1B, upper panel). Subsequent subpassage of these cells for 60 days in medium supplemented with 10% AL rat serum resulted in dramatic morphological changes, such as enlarged and flattened cell shapes as well as low saturation densities, both typical manifestations of senescence (Fig. 1B, lower left panel). In contrast, CR rat serum-treated cells during the same time period retained fibroblastic morphology and attained higher saturation densities, indicating that CR serum was able to delay the passage-induced senescent phenotype (Fig. 1B, lower right panel).

### CR serum treatment reduces SA-β-gal activity in normal human diploid fibroblasts

**Figure 2:**
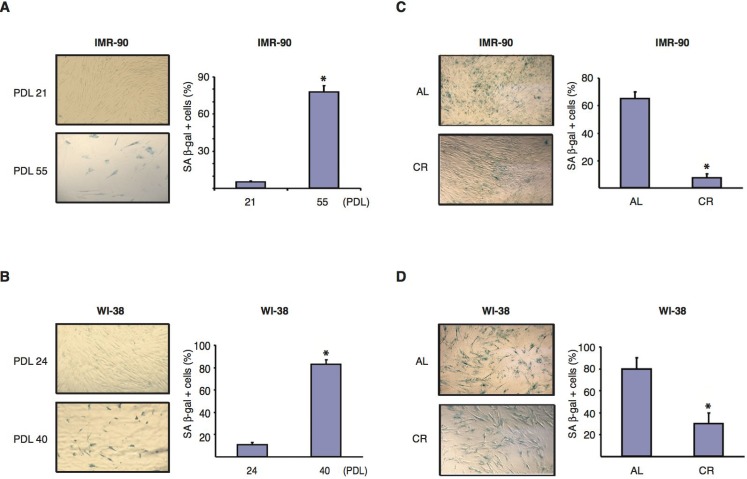
CR serum reduces SA-β-gal activity in normal human diploid fibroblasts (**A** and **B**) Representative photomicrographs of SA-β-gal staining (left panels) of IMR-90 fibroblasts at PDL 21 and 55 (**A**) and of WI-38, fibroblasts at PDL 24 and 40 (**B**). Data were obtained from cells cultured in medium supplemented with 10% FBS. Original magnification: X100. Graphs on the right show the average percentage of β-gal positive cells shown in left panels, and represent the average -/+ SEM from three independent experiments. * = statistically significant (p<0.05) for early vs. late passage. (**C** and **D**) Representative photomicrographs of SA-β-gal staining (left panels) of IMR-90 (PDL37) fibroblasts (**C**) and WI-38 (PDL36) cells (**D**) cultured for one week in the presence of AL (upper panels) or CR serum (lower panels). Original magnification: X100. Graphs on the right show the average percentage of β-gal positive cells shown in left panels. Data represent the average -/+ SEM from three independent experiments. * = statistically significant (p<0.05) CR vs. AL.

Senescent cells contain elevated levels of senescence-associated β-galactosidase (SA β-gal), an endogenous β-galactosidase that is active at pH 6.0 [47]. To determine whether CR serum affected the level of SA β-gal activity in normal human diploid fibroblasts, IMR-90 and WI-38 cells were grown in the presence of various sera, fixed at different passage numbers and stained with a β-gal substrate for microscopic analysis. As shown in Figure 2A, IMR-90 fibroblasts cultured in normal DMEM medium supplemented with 10 % FBS displayed a 15.8 fold increase in the percentage of SA β-gal-positive cells from PDL 21 to PDL 55. Similarly, the percentage of SA β-gal-positive WI-38 fibroblasts cultured in DMEM supplemented with 10% FBS showed an 8.3 fold increase from PDL 24 to PDL 40 (Fig. 2B). When IMR-90 cells grown in the presence of rat sera were analyzed, we found that at PDL 37, the percentage of SA β-gal-positive fibroblasts cultured in DMEM containing 10 % CR serum was reduced more than 8 folds compared to that of cells of the same passage number grown in 10% AL serum (Fig. 2C). A similar but more modest effect was also observed in WI-38 cells at PDL 36 (Fig. 2D). Thus, CR serum was able to significantly reduce the amount of passage-induced SA-β-Gal activity in two independent lines of normal human diploid fibroblasts.

### CR serum treatment significantly decreases the senescence-associated activation of MMP-2 in normal human diploid fibroblasts

**Figure 3:**
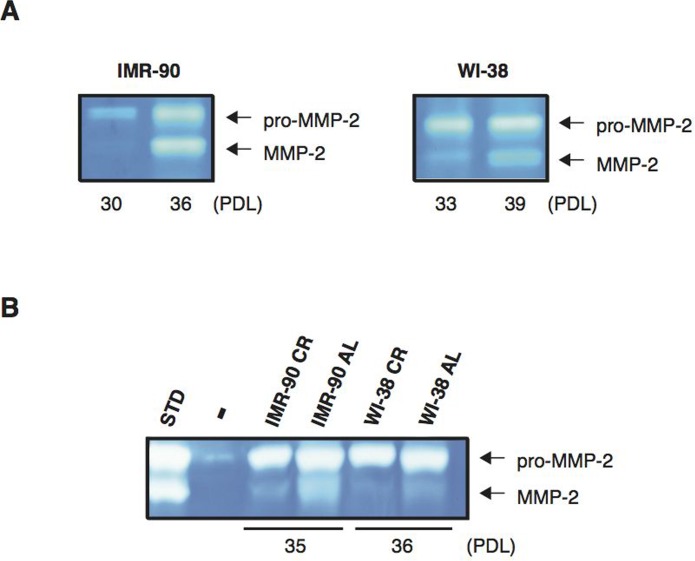
CR treatment reduces MMP-2 activity in normal human diploid fibroblasts (**A**) Gelatin zymograms of protein extracts from IMR-90 cells at PDL 30 and 36 (left panel), and from WI-38 cells at PDL 33 and 39 (right panel) are shown. These cells were cultured in DMEM with 10% FBS prior to processing. (**B**) Gelatin zymograms of protein extracts from IMR-90 at PDL 35 and WI-38 at PDL 36 that were cultured in the presence of sera from AL or CR rats, as indicated, are shown. The unprocessed pro-form of MMP-2 (pro-MMP-2) and the mature, cleaved form of MMP-2 (MMP-2) are indicated. MMP-2 standard control (STD) and negative control sample (−) are shown.

Cells undergoing replicative senescence contain elevated levels of secreted matrix metalloproteinase type-2 (MMP-2) activity. Full enzymatic activity of MMP-2 requires that the enzyme be cleaved by membrane-tethered MT-MMPs, in particular membrane type-1 matrix metalloproteinase (MT1-MMP)[48–51]. To assess the effects of CR serum on the activation of MMP-2, we performed gelatin zymography studies. As shown in Figure 3A, increasing passage numbers of normal human fibroblasts in DMEM 10 % FBS led to a significant increase in MMP-2 processing and activity in both IMR-90 cells (passaged from PDL 30 to PDL 36; left panel) and WI-38 cells (passaged from PDL 33 to PDL 39; right panel). When the levels of MMP-2 activity in IMR-90 (PDL 35) fibroblasts grown in medium containing either rat AL or CR serum were analyzed, we found that CR treatment significantly reduced MMP-2 activation Fig. 3B). Similar results were observed when WI-38 (PDL 36) cells exposed to AL or CR serum were examined Fig. 3B).

### SIRT1 protein expression is downregulated in normal human fibroblasts during senescence and CR serum-treatment delays this effect

**Figure 4:**
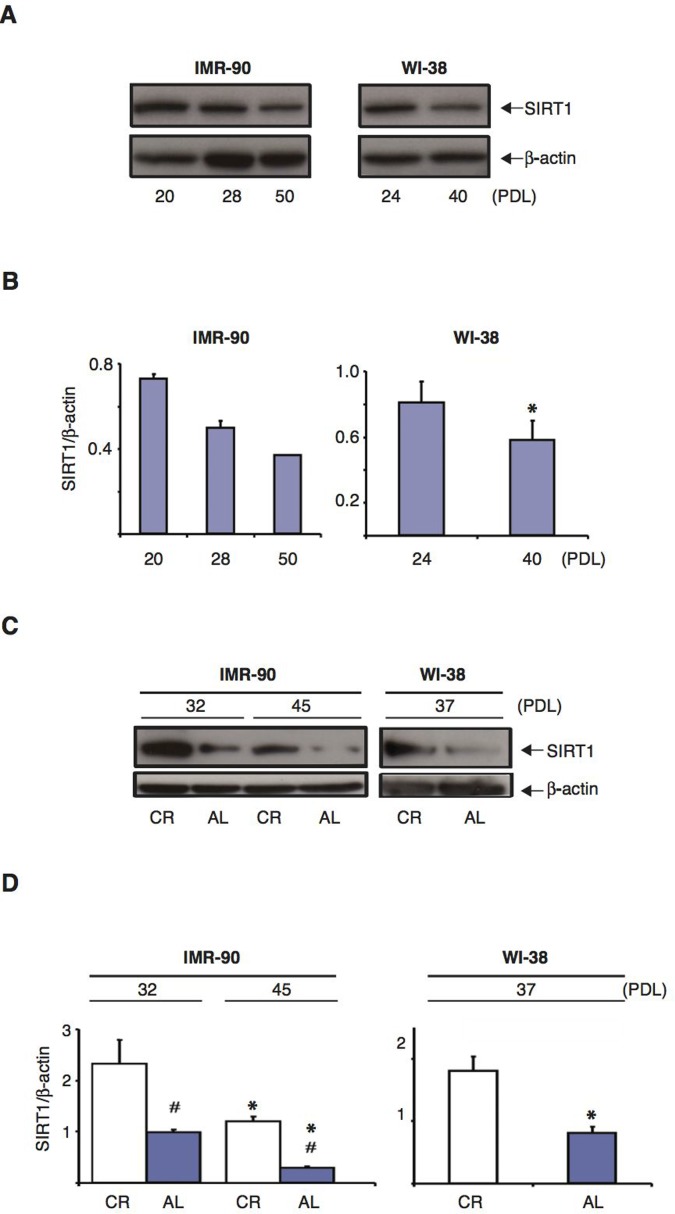
SIRT1 protein levels in normal human diploid fibroblasts decrease with increasing passage number and CR treatment retards this effect (**A**) Representative immunoblots for SIRT1 (upper panels) and β-actin (lower panels) proteins from IMR-90 cells (left panel) and WI-38 cells (right panel) at different passage number. (**B**) Quantification of SIRT1 protein levels shown in (**A**), after correction for β-actin loading controls. Data represent the average -/+ SEM from three independent experiments; *= statistically significant (p<0.05). (**C**) Representative immunoblots of SIRT1 (upper panels) and β-actin (lower panels) proteins from IMR-90 cells at PDL 32 and PDL 45 (left panels) and WI-38 cells at PDL 37 (right panels) cultured for one week in media containing 10% CR or AL serum. (**D**) Quantification of SIRT1 protein levels shown in (C), after correction for β-actin loading control. Data represent the average -/+ SEM from three independent experiments (*and #= statistically significant (p<0.05) for PDL effect and CR vs. AL effect, respectively).

At the cellular level, CR treatment is believed to enhance resistance to various forms of stress at least in part via the upregulation of sirtuins [38,52–54]. Since CR serum had such a profound effect on the development of a senescent phenotype in human fibroblasts (Figs. 1 and 2), we first analyzed the levels of SIRT1 protein in IMR-90 and WI-38 cells at different passage numbers to determine whether its expression was modulated during the senescence process. As shown in Figures 4A and B, the amount of SIRT1 protein in IMR-90 fibroblasts grown in the presence of 10% FBS progressively declined with increasing cumulative PDL (left panels). Specifically, between PDL 20 and PDL50 cells underwent a dramatic 50% reduction in SIRT1 protein levels. Similarly, the amount of SIRT1 in WI-38 fibroblasts was significantly decreased from PDL 24 to PDL 40 (Fig.4A and B, right panels). These results indicate that as human fibroblasts are passaged *in vitro* and progress toward replicative senescence, SIRT1 protein expression is significantly downregulated.

IMR-90 fibroblasts grown in the presence of 10% rat AL serum also showed a marked decline in SIRT1 levels from early (PDL 32) to late (PDL 45) passages Fig. 4C and D, left panels). Interestingly, despite the fact that IMR-90 fibroblasts grown in the presence of 10% rat CR serum also experienced a reduction in SIRT1 levels from PDL32 to PDL45, the overall SIRT1 protein levels in these cells were much higher than those observed in AL serum-treated controls. Specifically, the amount of SIRT1 protein found in IMR-90 fibroblasts grown in the presence of CR serum at PDL45 was 15-20% higher than that present in IMR-90 fibroblasts grown with AL-serum at PDL32 Fig. 4C and D, left panels). CR rat serum also preserved SIRT1 protein levels in WI-38 fibroblasts when compared to AL rat serum treatment at an intermediate (PDL 37) passage number Fig. 4C and D, right panels). These results indicate that CR serum treatment significantly prevents the senescence-associated SIRT1 downregulation displayed by normal human fibroblasts *in vitro*.

### Senescence in normal human fibroblasts is delayed by SIRT1 overexpression

**Figure 5:**
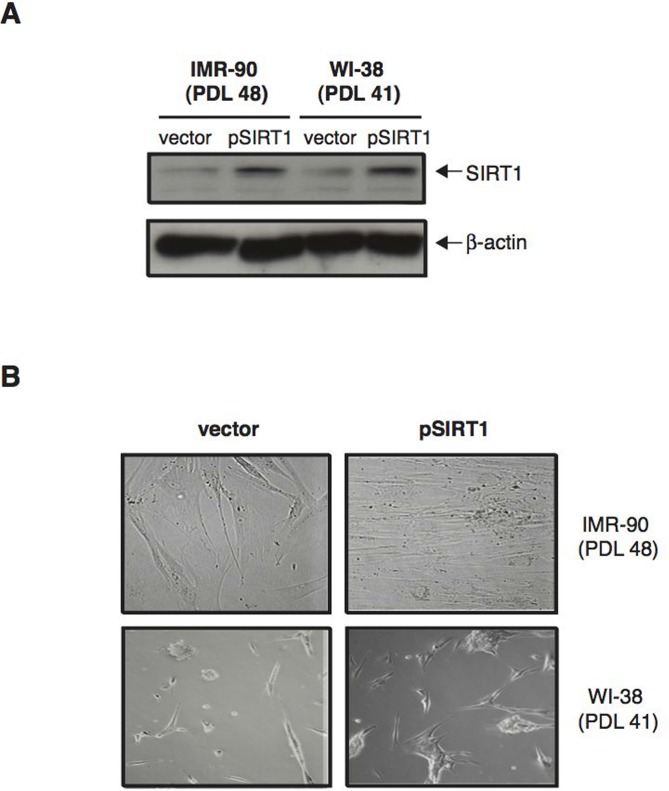
Over-expression of SIRT1 in normal human diploid fibroblasts delays senescence (**A**) Representative immunoblots for SIRT1 (upper panel) and β-actin (lower panel) proteins from IMR-90 (PDL 48) and WI-38 (PDL 41) cells 24 hours after transfection with either pSIRT1 (second and fourth lane) or a control plasmid (vector; first and third lanes) and cultured in medium supplemented with 10 % FBS. (**B**) Representative phase-contrast photomicrographs of IMR-90 cells (PDL 48) and WI-38 cells (PDL 41) two weeks after transfection with either pSIRT1 or vector control. Original magnification: X200.

The correlation between increased SIRT1 levels and senescence retardation displayed by normal human diploid fibroblasts treated with CR-serum suggested that SIRT1 may play an important role in this process. To investigate the effects of SIRT1 modulation on senescence in these cells, we first induced SIRT1 overexpression in IMR-90 and WI-38 fibroblasts at a late passage number, and analyzed cells for the development of a senescent phenotype. To this end, IMR-90 (PDL 48) and WI-38 (PDL 41) cells grown in DMEM supplemented with 10% FBS were transfected with either a plasmid coding for SIRT1 (pSIRT1) or a control empty plasmid (Vector). As shown in Figure 5A, twenty-four hours after transfection, SIRT1 levels were significantly increased in pSIRT1-transfected cells compared to vector-transfected controls. Remarkably, after two weeks in culture, IMR-90 and WI-38 fibroblasts overexpressing SIRT1 displayed a significantly higher cellular density than control cells Fig. 5B). Thus, overexpression of SIRT1 in late passage human fibroblasts grown in medium containing FBS resulted in delayed senescent growth arrest.

### Senescence in normal human fibroblasts is accelerated by siRNA-induced downregulation of SIRT1 and CR serum partially rescues this effect

**Figure 6:**
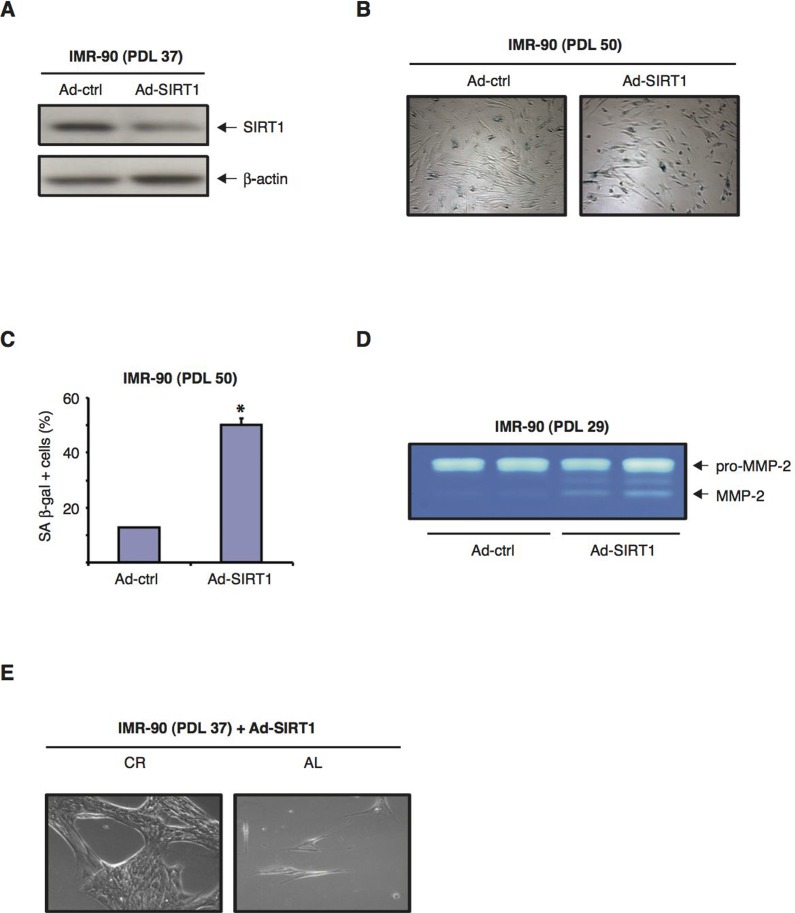
SIRT1 siRNA-mediated downregulation exacerbates the development of senescence in normal human diploid fibroblasts (**A**) Representative immunoblots for SIRT1 (upper panel) and β-actin (lower panel) proteins from IMR-90 cells (PDL 37) forty-eight hours after infection with either Ad-SIRT1 or an empty adenoviral vector as negative control (Ad-ctrl). (**B**) Representative phase-contrast photomicrographs showing SA β-gal staining of IMR-90 cells at PDL 50 forty-eight hours post-infection with either Ad-SIRT1 or Ad-ctrl (negative control). Original magnification: X100. (**C**) Quantification of the percentage of SA β-gal positive cells shown in (**B**).( **D**) Representative zymograms from early passage IMR-90 (PDL 29) cells seventy-two hours after transfection with Ad-SIRT1 siRNA or Ad-ctrl vector. Representative data from three independent experiments are shown. (**E**) Phase-contrast (photomicrographs of IMR-90 at PDL 37 infected with Ad-SIRT1, and subsequently incubated for 72 hours in media containing 10% of either CR (left panel) or AL (right panel) rat serum. Original magnification: X200.

We then tested whether downregulation of SIRT1 levels in normal human fibroblasts could accelerate cellular senescence. To this end, IMR-90 cells were infected with Ad-SIRT1-siRNA, an adenoviral vector that has been shown to efficiently knockdown SIRT1 gene expression [38]. Immunoblot analysis showed that forty-eight hours after Ad-SIRT1-siRNA infection of IMR-90 (PDL 37), approximately 80% of the SIRT1 protein was downregulated when compared to SIRT1 levels in cells infected with an empty adenoviral control Fig. 6A). Similar results were observed with later passage IMR-90 (PDL 50) fibroblasts (data not shown). Interestingly, after 72 hours in culture in DMEM 10% FBS, the percentage of senescent IMR-90 (PDL 50) cells infected with Ad-SIRT1-siRNA was approximate-ly 4-fold higher than that of cells infected with the control adenoviral vector, as measured by SA-β-GAL expression Fig. 6B and C). Interestingly, the knock-down of SIRT1 in early passage IMR-90 cells (PDL 29) led to a significant increase in MMP-2 activation Fig. 6D). These results indicate that reduction of SIRT1 protein levels alone can have a significant impact on the development of senescence manifestations in normal human fibroblasts.

When low passage IMR-90 cells (PDL 37) infected with Ad-SIRT1-siRNA were subsequently exposed to medium containing 10% CR rat serum for 72 hours, their growth rate was improved (i.e. they attained higher cellular density) compared to that displayed by the same cells treated with medium containing 10 % AL serum Fig. 6E). Therefore, even in the context of siRNA-induced SIRT1 downregulation, CR serum treatment still to some extent retarded the development of a senescent phenotype in normal human fibroblasts.

### Modulation of SIRT1 levels by either over-expression, knockdown or CR-treatment is associated with altered p53 levels

**Figure 7:**
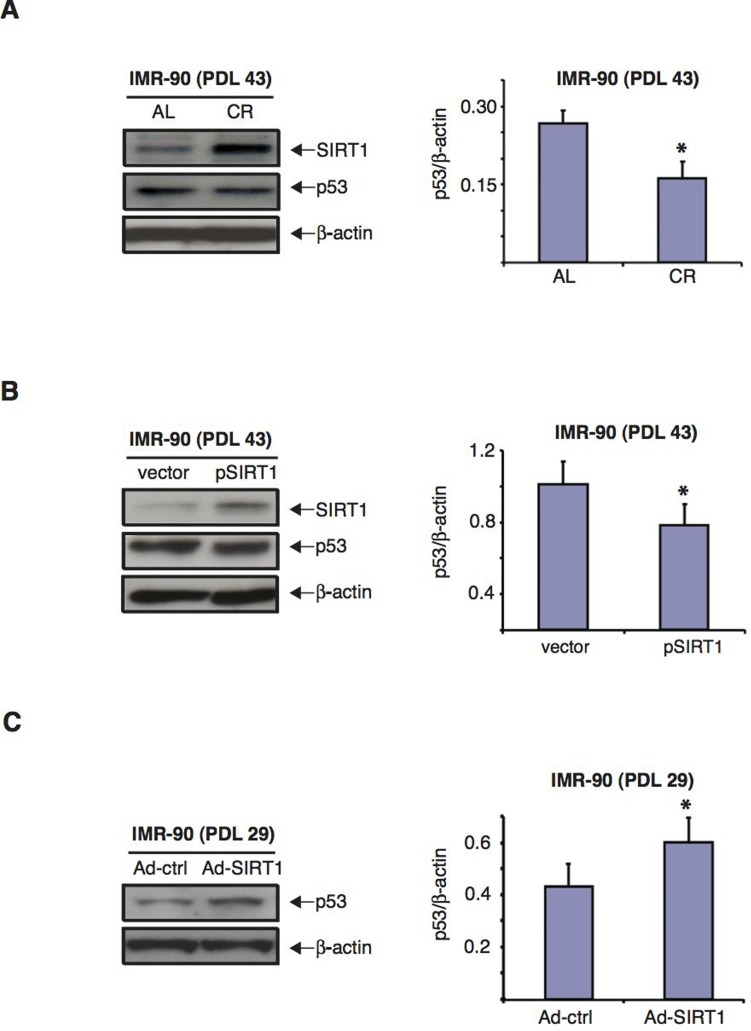
CR serum and over-expression of SIRT1 decrease p53 levels whereas SIRT1 knockdown increases them in IMR-90 cells (**A**) Representative immunoblots for SIRT1 (upper panel), p53 (middle panel) and β-actin (lower panel) proteins of cell extracts from IMR-90 cells (PDL 43) cultured for 72 hours in media with CR (second lane) or AL (first lane) rat serum. Right panel shows the quantification of p53 levels for immunoblots shown on the left after correction for actin controls (* = p< 0.05 CR vs. AL). (**B**) Representative immunoblots for SIRT1 (upper panel), p53 (middle panel) and β-actin (lower panel) proteins of extracts from IMR-90 cells (PDL 43) transfected twenty four hours prior to lysis with either pSIRT1 or empty vector. Right panel shows the quantification of p53 levels for the immunoblots shown on the left after correction for actin controls (* = p<0.05, pSIRT1 vs. vector). (**C**) Representative immunoblots for p53 (upper panel) and β-actin (lower panel) of proteins extracts from IMR-90 cells (PDL 29) forty-eight hours after infection with either Ad-SIRT1 or Ad-ctrl (negative control). Data shown in **A**, **B** and **C** are representative of three independent experiments per group.

The tumor suppressor p53 signaling pathway plays a major role in the development of senescence [3,8,55,56], and it has been reported that SIRT1 can modulate cellular stress responses and survival through regulation of p53 [38,57–60]. Since CR treatment delayed senescence and efficiently preserved SIRT1 expression in IMR-90 human diploid fibroblasts with increasing cumulative PDLs, we tested whether p53 levels were also modulated by CR treatment in these cells. As shown in Figure 7A, we found that CR serum treatment of IMR-90 fibroblasts at PDL43 was accompanied by a decrease in p53 protein levels when compared to AL serum treatment. Specifically, the amount of p53 present in cells cultured in medium with CR serum was 1.6-fold lower than that of the same cells cultured in medium with AL serum (Fig.7A, right panel). Overexpression of SIRT1 in pSIRT1-transfected IMR-90 (PDL 43) fibroblasts grown in normal medium caused a significant decrease in p53 expression levels (Fig.7B). Conversely, the knockdown of SIRT1 in early passage IMR-90 (PDL 29) fibroblasts enhanced p53 expression (Fig. 7C).

## DISCUSSION

Several physiological consequences of CR regimes, including reduced cellular proliferation and increased stress resistance in certain cell types, can be reproduced *in vitro* by culturing cells with serum obtained from animals that were fed CR diets. For instance, FaO cells (a transformed rat hepatoma cell line) and rat primary hepatocytes exposed to CR sera from either rats or Rhesus monkeys have been shown to display enhanced responsiveness to stresses such as heat shock-induced toxicity and oxidative stress by hydrogen peroxide (H2O2) [44]. A similar observation was recently made with sera collected from human participants of the FEAST study, which induced a form of CR by alternate-day fasting or ADF (i.e. short, regular intervals of complete caloric deprivation). The study compared the effects on human hepatoma HepG2 cells of sera collected from individuals at baseline (before dieting) versus sera collected from the same individuals at the end of the dieting period [53]. Interestingly, cells cultured in sera from participants following the ADF regime showed decreased proliferation and increased resistance to heat shock stress.

Other consequences of CR regimes, like their beneficial effects on the cardiovascular system, appear to be accompanied by cellular proliferation. Csiszar et al. reported that human endothelial cells cultured with sera derived from *Macaca mulatta* on long-term CR displayed increased migration, proliferation and formation of capillary-like structures when compared to cells treated with sera derived from ad-libitum-fed control monkeys [61]. These effects were correlated with upregulated vascular endothelial growth factor (VEGF) signaling, and suggest that increased angiogenesis caused by circulating serum factors could be a potential mechanism by which CR improves cardiac function and prevents vascular cognitive impairment *in vivo*.

While all of the reported CR-mediated effects *in vitro* have been observed after incubation of cells for short periods with CR sera from various species, little is known about the long-term effects of CR serum treatment in cultured cells. An important cellular consequence of the process of ageing is replicative senescence, whereby cells lose their replicative capacity and irreversibly exit the cell cycle. Decreased senescence *in vivo* is believed to contribute to delayed ageing and the increased tolerance to stress observed in organisms subjected to CR regimens [3,8]; however, the study of this cellular process *in vivo* (either in animals or humans) is experimentally challenging. Therefore, we decided to test the effects of CR serum on cellular senescence *in vitro* using normal human diploid fibroblasts, which undergo replicative senescence after several passages in culture. Our results clearly show that CR serum can significantly delay senescence progression of these cells, resulting in increased replicative lifespan. This was exemplified by delayed appearance of senescent morphological changes, decreased levels of p53 and reduced activity of senescence markers such as SA β-galactosidase and MMP-2, with the consequent increased in cumulative PDL (Figs. 1, 2, 3 and 7).

The molecular mechanisms mediating the health benefits associated with CR are not fully understood, and while some may be shared among different target organs and cell types, others are likely to be cell-type specific. For instance, it was recently reported that CR conferred persisting anti-oxidative, pro-angiogenic, and anti-inflammatory cellular effects in rat cerebro-microvascular endothelial cells, in part by modulating the levels and activities of Nrf2, NFkB, and miR-144 [62]. Among the most ubiquitous molecular targets of CR are the sirtuin proteins, whose levels are increased in all animal models of CR, suggesting these enzymes play a major role in CR-induced physiologic activities. Studies performed in yeast, flies, worms and mice have shown that increased sirutin activity due to either protein over-expression or treatment with sirtuin-activating compounds such as resveratrol, can *per se* recapitulate several of the effects mediated by CR [33–37,41–43]. Importantly, the CR-associated SIRT1 increases observed in animals can be recapitulated in *in vitro* models of CR. CR-sera from rats and monkeys as well as from human participants in the FEAST study have all consistently induced increased levels of SIRT1 in various cell types exposed to these sera for short time periods [38,53]. In human cells, CR serum-mediated induction of SIRT1 leads to the sequestration of proapoptotic factors and consequent promotion of cell survival [38]. Here, we show that SIRT1 levels decline with passage number *in vitro* as IMR-90 and WI-38 normal human fibroblasts progress toward replicative senescence (Fig. 4A and B). This is in accordance with previous reports showing reduced SIRT1 levels in senescent human and mouse fibroblasts, human smooth muscle cells (SMC) as well as human endothelial cells [63–66]. Interestingly, when IMR-90 and WI-38 cells were repeatedly passaged in the presence of CR rat serum they displayed much higher levels of SIRT1 than control cells passaged in AL serum (Fig. 4C and D). Therefore, CR serum protects cells from SIRT1 decreases associated with the senescence process throughout long time periods. Furthermore, experiments where SIRT1 levels were experimentally manipulated at different passage numbers suggested that this protein is important for the proper execution of the senescence program (i.e. triggering it when eliminated by siRNA-knockdown and delaying it when over-expressed) (Figs. 5 and 6). Previous studies testing the ability of SIRT1 to delay senescence and increase replicative lifespan have produced conflicting results. Over-expression of SIRT1 in mouse embryo fibroblasts and articular chondrocytes has been reported to inhibit senescence [67,68], whereas reduced SIRT1 activity has been shown to induce senescence in human breast cancer MCF-7 cells, endothelial cells and articular chondrocytes [64,65,68]. However, others have found that SIRT1 over-expression does not affect the replicative lifespan of human fibroblasts [69]. Importantly, mouse embryonic fibroblasts from SIRT1-deficient animals displayed increased rather than decreased replicative lifespan [70]. Ho *et al.* have suggested that some of these differences may be explained by different levels of activity of the NAD+ salvage pathway under various experimental paradigms [63]. These authors observed that in SMCs, SIRT1 over-expression conferred significant extension of replicative lifespan only when the activity of the nicotinamide phosphorybosiltransferase (Nampt), a rate-limiting enzyme for NAD+ salvage, was also increased [63]. Nampt activity has been shown to decline as cells progress toward senescence [71–75]. Therefore, it is possible that in IMR-90 and WI-38 cells the basal activity of Nampt (and/or other NAD+-generating enzymes) is sufficiently high to allow SIRT1-mediated senescence modulation and/or that the sera tested in our study contain factors that can enhance their function. Future experiments should test these possibilities. We observed that CR serum-treatment of early passage IMR-90 cells undergoing siRNA-induced SIRT1 downregulation (and therefore accelerated senescence) still had some retarding effect regarding the appearance of a senescent phenotype Fig. 6E). This result suggests that in addition to elevating SIRT1 levels, CR treatment exerts a wider effect on senescence progression in these cells. Whether results obtained with this *in vitro* system are a reflection of the modulation of senescence in organisms undertaking CR diets remains to be determined.

Additional effects of CR on the senescence program likely include the nutrient sensor mammalian target of rapamycin (mTOR), which is implicated in stimulating cell growth and an array of cellular functions [76, 77]. Nutrients as well as insulin and other growth factors activate mTOR, which can lead to accelerated cellular senescence [78, 79, 80]. Conversely, Rapamycin has been shown to delay cellular senescence through the inhibition of mTOR [81, 82]. Interestingly, Resveratrol can suppress cellular senescence at least in part by antagonizing the mTOR pathway through the activation of Sirt1 [83, 84]. CR serum has less nutrient availability and leads to elevated levels of Sirt1, a combination that would be expected to inhibit mTOR signaling [85].

Responses to CR treatment are expected to vary vastly between normal and cancerous cells. For instance, cancer cells typically show sustained aerobic glycolysis, a phenomenon known as the Warburg effect, and therefore are likely to be much more sensitive to nutrient deprivation during CR than normal cells [86]. Under certain experimental paradigms, cancerous cells also appear to display higher sensitivity than normal cells to CR-mediated reductions in the levels of circulating factors like Insulin-like growth factor 1 (IGF-1) [87, 88]. This increased sensitivity, at least in tumors of epithelial origin, appears to be dependent on the activity of the phosphoinositide 3-kinase (PI3K)/AKT signaling pathway [89, 90]. Differences in the activities of several other signaling molecules, including mTOR and Sirtuins, have also been implicated in the differential effects of CR on normal versus tumor cells [91].

Abrogation of programmed senescence appears to be a fundamental prerequisite for tumor formation [3,7,9,19]. In that regard, the senescence-suppressing effects of CR would be predicted to have tumorigenic activity; however, long term CR in various animal models has been consistently associated with lower cancer risk [92–97]. Clearly, other beneficial effects of CR must operate to prevent cancerous changes in long-lived cells. On the other hand, and despite the predicted anti-tumor activity of replicative senescence, this process might also contribute to tumor formation and spread under certain circumstances (e.g. through the microenvironmental disruption that results from the secretion of extracellular matrix-degrading proteases by senescent cells). The expression of MT1-MMP and the activation of MMP-2, for instance, strongly correlate with tumor growth, neovascularization, and metastasis [98–108]. In addition, MMP-2 protein levels have been shown to increase in all tumor-derived fibroblast lines [17,109]. Our data show that whereas normal senescent human diploid fibroblasts produced high levels of MMP-2, CR serum treatment led to a significant reduction in MMP-2 activity associated with passage number Fig. 3). Whether this effect is representative of *in vivo* consequences of CR and could contribute to its anti-tumor activity remains an interesting possibility. The reduction in MMP-2 displayed by CR-treated human diploid fibroblasts in this study correlated with increased SIRT1 levels, and knockdown of SIRT1 in early passage cells enhanced MMP-2 activation to levels comparable to those associated with cells at a much later passage number (i.e. senescent) (Fig. 6D). These data highlight the potential beneficial effect of interventions based on CR or SIRT1-agonists like resveratrol against tumor-promoting MMP-2 activity.

SIRT1 has been shown to regulate cellular responses to diverse stresses in part through deacetylation of the tumor-suppressor p53 and the consequent down-regulation of its transcriptional activity in various cell types, including neurons, cardiac myocytes, and smooth muscle cells [110–118]. Several lines of evidence show that p53 also plays a central role in the progression of cellular replicative senescence. Elimination of p53 activity from pre-senescent cells through sequestration by viral oncoproteins extends the lifespan of human cells by as much as 200 population doublings [119,120]. Similarly, introduction of dominant negative versions of mutant p53 also increases cellular lifespan [91] and micro-injection of anti-p53 antibodies into senescent human fibroblast reinitiates DNA synthesis and allows further population doublings [121]. Our data indicate that CR-mediated modulation of SIRT1 signaling in human diploid fibroblasts directly correlated with decreased expression levels of p53 (Fig. 7). Once again, how a treatment like CR can lead to both lower p53 levels and reduced cancer risk remains to be elucidated. Manipulation of SIRT1 levels by silencing or overexpressing SIRT1 also resulted in increased and decreased p53 levels, respectively (Fig. 7). A recent report showed that normal human fibroblasts *in vitro* and colon adenomas *in vivo* displayed a particular senescence-associated signature of p53 isoform expression, with elevated levels of p53β and reduced ß133p53 [56]. Whether CR treatment results in altered expression of any of these isoforms should be investigated.

In summary, this study demonstrates that delayed senescence onset, one of the anti-ageing effects believed to be exerted by CR, can be induced *in vitro* in cells incubated in media supplemented by serum collected from animals fed a CR diet. Specifically, exposure of normal human diploid fibroblasts to CR rat serum compared to AL rat serum resulted in i) sustained increases in SIRT1 levels, ii) decreased p53 levels, iii) delayed morphologic senescent changes, iv) reduced activities of SA β-gal and MMP-2, and v) increased replicative lifespan. The modulation of cellular senescence by CR serum described here further validates the use of this *in vitro* model to investigate the mechanisms behind the beneficial effects of CR. We now have an experimental system to identify CR serum components or CR-mimetic compounds that regulate senescence and to study the molecular consequences of their effects. We also show that manipulation of SIRT1 levels by either over-expression (mimicking the effects of CR serum) or knockdown of this protein (mimicking the effects of AL serum) resulted in delayed and accelerated cellular senescence, respectively. Taken together, these findings indicate that the modulation of the SIRT1 signaling pathway *per se* can have a significant impact on the progression of the senescence program in normal human fibroblasts, and suggest that SIRT1 plays an important role in CR-mediated senescence retardation and replicative lifespan extension in these cells.

## MATERIALS AND METHODS

### Animals, Dietary Manipulations and Sera

Male Fisher-344 rats fed either an AL or CR diet were used in these experiments. AL animals were fed a NIH-31 standard diet while CR animals were given a vitamin- and mineral-fortified version of the same diet. CR animals were in a 60% calorie restriction since weaning (i.e. 40% less food (by weight) than the average AL consumption). Water was available ad libitum for both groups. Animals were maintained under controlled conditions including 12 on/12 off light cycle, with appropriate temperature and humidity. The animal protocol used was approved by the Institutional Animal Care and Use Committee of the Gerontology Research Center and complied with the guide for the care and use of laboratory animals (NIH publication No. 3040-2, revised 1999).

Sera from these animals were collected every other week during a 6-month period. All sera were obtained from fasted, anesthetized animals. Rats were anesthetized and a 21-gauge catheter was inserted into the tail vein. 1.5 ml of whole blood was then collected and allowed to clot for 20-30 minutes, followed by centrifugation for 20 min at 2500 rpm. Sera were removed from the centrifuged samples and stored frozen until used. All sera utilized were thawed and heat inactivated at 56 ^o^C prior to use in cell culture experiments.

### Cell Culture

IMR-90 (I90-79), IMR-90 (I90-26) and WI-38 (AG06814-N) with PDL at freeze of 17, 30 and 15, respectively, were purchased from Coriell Institute for Medical Research (Camden, NJ). These cells were grown in Minimum Essential Medium (MEM) (1X), with 2mM L-glutamine, 100 μg/ml penicillin, and 100 μg/ml streptomycin and supplemented with either 10% fetal bovine serum (FBS) or 10% serum from AL- or CR-fed rats. Cells were maintained at 37°C under a humidified 5% CO_2_ and 95% O_2_ air atmosphere. Except for experiments describing Figure 1, IMR-90 (I90-79) cells were utilized for all other experiments.

### Transfections and Infections

The pSIRT1 expression vector, adenovirus SIRT1 siRNA (Ad-SIRT1), and their respective negative controls were kindly provided by Dr. D. Sinclair (Harvard University) and have been previously described [38]. Cells were transfected with pSIRT1 or control plasmid (empty vector) using Lipofectamine 2000 (Invitrogen, Carlsbad, CA) following manufacturer's instructions. Adenoviral infections with either Ad-SIRT1 or Ad-control were carried out at a multiplicity of infection (MOI) of 100, as previously described [38].

### SA-β-galactosidase Assay

The percentage of SA-β-gal-expressing cells was determined as previously described [47]. Briefly, cells in 6-well plates were washed twice in PBS, fixed for 5 min in 4% paraformaldehyde in PBS and washed three times in PBS. The cells were then incubated overnight at 37 °C with fresh SA-β-gal staining solution (1 mg of [5-bromo-4-chloro-3-indolyl β-d-galactopyranoside]/ml of 5 mM potassium ferrocyanide, 5 mM potassium ferricyanide, 150 mM NaCl, 2 mM MgCl_2_ in 40 mM citric acid/sodium phosphate, pH 6.0) and examined under the microscope.

### Western blotting

Whole-cell lysates were prepared by scraping cells in Laemmli buffer (0.12 M Tris, pH 6.8, 4% (w/v) SDS, 20% (v/v) glycerol) containing a protease inhibitor cocktail (Sigma, St. Louis, MO). Proteins were then separated by SDS/PAGE under reducing conditions on a 12% gel and transferred to PVDF membranes. Unspecific binding was blocked by incubation in 5% milk blocking buffer (PBS, 5% nonfat milk and 0.1% Tween 20). Membrane bound proteins were then immunoblotted with antibodies to p53 (ab7757-100, abcam), actin (Santa Cruz Biotech, Santa Cruz, CA), or Sirt1 (Millipore, Billerica, MA). Signals were developed using ECL reagent (Amersham Pharmacia Biotech, Buckinghamshire, England) and densities of the bands were evaluated using a Syngene Gene Genius Bio-Imaging System (Imgen, Alexandria, VA). Protein loading was evaluated either using b-actin antibody (Santa Cruz) or Ponseau S staining (Sigma-Aldrich).

### Gelatin zymography

Gelatin zymography was performed as previously described with some modifications [48]. Briefly, cells were lysed in SDS-sample buffer and aliquots (equivalent to 10^5^ cells per lane) were loaded without reduction onto 10% Novex gelatin zymogram gels (Invitrogen), run at 125 V for 90 minutes, and incubated in Novex Zymogram renature buffer for 30 minutes (to remove SDS and renature the MMP-2 species). Then the gels were incubated in developing buffer at 37^o^ C for 4 hours to induce gelatin lysis by renatured MMP-2.

### Statistical Analysis

All results are expressed as the mean ± SEM. Statistical comparisons for multiple group differences were made via a one-way or two-way ANOVA, followed by Bonferoni post hoc tests; and for two group comparisons were determined via Student's t-tests. A p value of < 0.05 was considered statistically significant.
